# Predicting and Visualizing Citrus Color Transformation Using a Deep Mask-Guided Generative Network

**DOI:** 10.34133/plantphenomics.0057

**Published:** 2023-06-07

**Authors:** Zehan Bao, Weifu Li, Jun Chen, Hong Chen, Vijay John, Chi Xiao, Yaohui Chen

**Affiliations:** ^1^ College of Informatics, Huazhong Agricultural University, Wuhan 430070, China.; ^2^ Engineering Research Center of Intelligent Technology for Agriculture, Ministry of Education, Wuhan, China.; ^3^ RIKEN, Guardian robot project, 2-2-2 Hikaridai Seika-cho, Sorakugun, 619-0288 Kyoto, Japan.; ^4^ Key Laboratory of Biomedical Engineering of Hainan Province, School of Biomedical Engineering, Hainan University, Haikou 570100, China.; ^5^ College of Engineering, Huazhong Agricultural University, 430070 Wuhan, China.

## Abstract

Citrus rind color is a good indicator of fruit development, and methods to monitor and predict color transformation therefore help the decisions of crop management practices and harvest schedules. This work presents the complete workflow to predict and visualize citrus color transformation in the orchard featuring high accuracy and fidelity. A total of 107 sample Navel oranges were observed during the color transformation period, resulting in a dataset containing 7,535 citrus images. A framework is proposed that integrates visual saliency into deep learning, and it consists of a segmentation network, a deep mask-guided generative network, and a loss network with manually designed loss functions. Moreover, the fusion of image features and temporal information enables one single model to predict the rind color at different time intervals, thus effectively shrinking the number of model parameters. The semantic segmentation network of the framework achieves the mean intersection over a union score of 0.9694, and the generative network obtains a peak signal-to-noise ratio of 30.01 and a mean local style loss score of 2.710, which indicate both high quality and similarity of the generated images and are also consistent with human perception. To ease the applications in the real world, the model is ported to an Android-based application for mobile devices. The methods can be readily expanded to other fruit crops with a color transformation period. The dataset and the source code are publicly available at GitHub.

## Introduction

Widely cultivated in more than 140 countries and regions, citrus is the highest-value fruit crop in the world, reaching an annual production of more than 48.4 million tons [1]. As the production growth is expected to gradually retard in the future [2], fruit quality and post-harvest processing become vital to further increase the profit margin for the industry. For most citrus species, the green color of the rind diminishes because of the presence of pigments [3], and other colors, such as yellow, orange, and red, emerge when carotenoids accumulate [4]. Because this process is strongly correlated to fruit development, citrus color is a good indicator of the maturity stage [5], and its proper monitoring enables better decisions of crop management practices. Moreover, the optimal flavor and storage life can only be obtained if the fruit is harvested at a specific maturity stage [6], and methods to predict citrus color transformation are therefore also important for the promotion of fruit quality and arrangement of the harvest schedule.

Currently, citrus color approximation and prediction are based on the experience of investigators, which lacks accuracy and is constrained to those with such capability. Advances in machine vision techniques have attracted a lot of attention because of their nondestructive and objective nature, and they have been successfully deployed for a variety of precision agriculture tasks including the characterization of plant varieties [7–11], robotic harvesting [12,13], and diseases diagnosis [14,15]. Jiménez-Cuesta et al. [16] have also attempted citrus color approximation by extracting color-based features from input images, with which classification or regression methods can be then performed [17]. However, the accuracy of color identification varies regarding different lightning conditions and view angles. It is also difficult for users to relate obtained color parameters, such as the standard citrus color index (CCI), to the corresponding visual characteristics and maturity stage. In more recent years, techniques using deep neural networks become trending owing to their ability to automatically learn and extract effective features, presenting more robust performance in complicated environments [18–20]. In one study, a multitask network architecture based on the Mask R-CNN framework was proposed to be directly used in orchards, in which it first located the citrus fruit in view and then evaluated its color and maturity stage [21].

Despite the aforementioned studies for citrus color monitoring, they focused on analyzing the color information of the citrus fruit only in the current image, and research to predict color transformation has been rarely reported. Moreover, instead of presenting a series of color parameters, techniques to visualize color transformation is also necessary as information on rind color can be more easily delivered to the users. The prediction of citrus color transformation via visualization can be defined as a conditional image generation problem, and generative adversarial networks (GAN) [22] are commonly used methods. Conditional GAN [23] achieved image generation conditioned on class labels. Isola et al. [24] proposed Pix2Pix for image-to-image translation and achieved reasonable results. Zhu et al. [25] proposed CycleGAN to address domain-to-domain problems such as collection style transfer, season transfer, and photo enhancement. In the work by Gatys et al. [26], the authors generated an image that has both the content of one image and the style of another image using a pixel iteration framework guided by a manual perceptual loss function. Johnson et al. [27] trained a generative neural network with Gatys’ perceptual loss function. Following Johnson’s work, mask-based style generation methods [28,29] acting on the local regions of images gradually emerged.

To ease the application of the algorithms, developing applications operating on edge devices is a common choice [30]. Relevant attempts usually accomplished model inferring based on cloud computing [31], which greatly reduces the computational costs on the hardware but requires a smooth internet connection. For the areas with unsatisfactory network signals, edge computing is needed to handle the tasks locally. As available edge devices still lack compute power and storage capability compared with cloud-based infrastructure [32,33], the algorithms need to be carefully designed to contrarian model parameters. However, generative model-based algorithms, which typically have more model parameters, have been rarely reported to be ported to edge devices in the agricultural domain.

Drawing inspiration from these studies, we presented a framework to both predict and visualize the color transformation of citrus fruit in orchards, with which an Android-based application was developed to further ease the application in the real world. The network model receives a citrus image taken in the orchard and the interested time interval *N*, and it is then able to output the image presenting the color of the same fruit after *N* days. While color transformation of the citrus rind is influenced by both time and environmental aspects, the latter are out of the scope of this study but can be considered by slight modifications of the network. Finally, we implement the proposed network with a user-friendly interface on an Android-based mobile device. The dataset and code have been provided publicly. The major contributions of this study include the following:

1. The dataset containing the images of 107 oranges captured throughout the color transformation period, which can be used as labeled samples for the training and validation of the network model;
2. A novel framework to predict and visualize color transformation of citrus fruit in the orchard based on a deep mask-guided generative network, which features easy data acquisition and high prediction accuracy;
3. A merged design of the network architecture that requires significantly reduced training and storage resources, enabling the implementation on mobile devices for wider applications.

The rest of this paper is organized as follows. The “Dataset” section introduces the dataset used in this paper, and the “Proposed framework” section states the details of the proposed framework. The “Results” section reports the experimental analysis of our approach, and discussion and conclusions are presented in the “Discussion” and “Conclusion” sections, respectively.

## Materials and Methods

### Dataset

#### Image acquisition

**Fig. 1.**
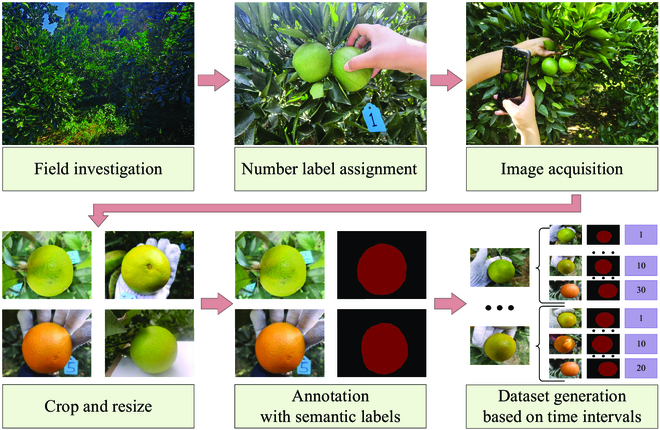
The process of building the dataset.

The process of building the dataset is shown in Fig. 1. Navel oranges were chosen as the sample fruit because of their long color transformation period, which helped ease image acquisition and analysis. Their rind color has also been reported to highly correlate to their maturity stages [6]. The images were acquired between 2021 November 15 and December 31 from 10:30 to 15:00 at the citrus orchard of Huazhong Agricultural University, Wuhan, Hubei Province, China. Thirty citrus trees at varying locations were first selected, among which 3 to 4 oranges without any external defects on each tree were then randomly chosen, resulting in a total of 107 sample oranges. A serial number label was assigned to each orange to distinguish these samples in the orchard. Image acquisition was initiated 30 d before the oranges reached the targeted color, and it was then performed every day from the front, bottom, and side views of each orange for continuous 45 d. All the images were captured in the natural environment. Although no restrictions were applied on the fruit position or background during image acquisition, a rough similarity in the illumination conditions was observed. The obtained images were resized to the resolution of 256 × 192. Because of weather conditions and some incorrect shooting operations, some images of the samples were missing. The number of images per sample ranged from 100 to 135. Images with severe shaking and ghosting were deleted to accurately annotate semantic information, and some severely blurred images with obvious color distortion were also removed for color prediction precision, resulting in the final dataset of 7,535 images. To enhance the robustness of the segmentation model, several additional images with excessive lighting conditions were captured and utilized during the training of the segmentation network.

#### Data annotation

Because we only focused on the color information of oranges in the generative model, the oranges were manually annotated with semantic labels using LabelMe. For each sample orange, we constructed a time interval mapping between the images and their date of acquisition. Subsequently, the dataset was partitioned into inputs and labels. The inputs contained the orange images collected at the current day and the interested time interval *N*(days), and the labels consisted of the corresponding images according to the above mapping and their semantic labels. To quantify the performance of the proposed model, the dataset including 107 samples was randomly divided into the training set, validation set, and test set with the ratio of 3:1:1, where the images belonging to the same sample only appeared in one of the sets. The validation set was used to select the appropriate hyperparameter, and the test set was assumed to be completely unknown data for performance evaluation. To further test the model performance and replicate the experiment, the dataset was also randomly and evenly divided into 5 sets for K-fold cross validation.

### Proposed framework

**Fig. 2.**
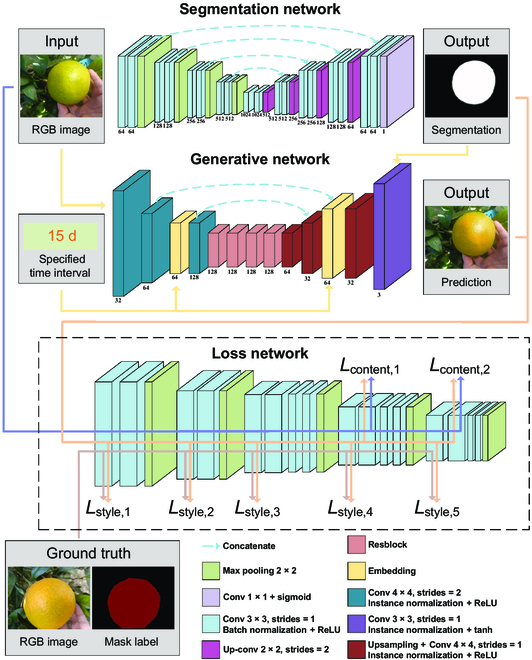
The architecture of the proposed deep mask-guided generative network.

As shown in Fig. 2, the proposed framework consists of 3 modules, a segmentation network, a generative network, and a loss network. The segmentation network is designed to identify the citrus fruit in view. Utilizing the segmented result, the generative network generates the future color of the fruit. The loss network measures the difference between the predicted color and true color, which can be used to update the parameters of the generative network. The combination of these 3 modules enables the network to predict the future color of the fruit based on the input image, and the predicted color can be generated on the local region where the fruit locates following the style of the original image.

#### Segmentation network and training

To locate the citrus fruit in view, we employ the U-Net encoder–decoder architecture because of its simplicity, efficiency, and robustness with limited amounts of data [34]. The size of the input image *x* is 256 × 192, and the encoder (left part) extracts image features using multiple 3 × 3 convolution layers and 2 × 2 max pooling operations with stride 2. These features are transferred to the decoder branch using the skip connection, and multiple skip connections at different encoder layers transfer the layer feature maps to the corresponding decoder layers. The decoder contains multiple decoding convolution layers and upsampling layers, and it concatenates the transferred encoding feature maps with the upsampled feature maps. The last layer of the decoder contains a sigmoid activation function, which generates the semantic segmentation of the fruit.

To avoid the situation where the denominator or numerator is 0, a modified Dice loss [35] is adopted for training the segmentation network as:

$$\text{Dice loss}=1-\frac{1+2\sum ‍p_{\mathrm{ij}}g_{\mathrm{ij}}}{1+\sum p_{\mathrm{ij}}^{2}+\sum ‍g_{\mathrm{ij}}^{2}}$$ (1)

where *p_ij_* and *g_ij_* represent the pixels with coordinate (*i*, *j*) in the predicted segmentation and ground truth, respectively.

#### Generative network

Similar to the segmentation network, the proposed generative network also adopts an encoder–decoder structure. The encoder is composed of multiple 4 × 4 convolution layers with stride 2 and a stack of identical 3 × 3 resblocks [36]. The decoder consists of several resize convolution layers and a 3 × 3 convolution layer, combined with the mask from the segmentation network to achieve background re-fusion. Furthermore, 2 trainable embedding layers are designed to encode the input time interval *N* to 2 64-dimension vectors, which are multiplied by the 64 feature maps from the second convolution layers in the encoder and decoder, respectively. All convolution layers are followed by instance normalization and activation function ReLU except the output layer, which uses a scaled tanh [37] to ensure the output pixels range in [0, 255].

The resize convolution layers double feature maps in height and width using the nearest neighbor interpolation; the results of which are then transferred to 4 × 4 convolution layers with stride 1. Instead of transposed convolution layers for upsampling, which may cause checkerboard effects [38], the resize convolution layers and skip connections contribute to acquiring images with higher quality. Through our design of encoder–decoder and embedding layers, the generated image $\hat{y}$ is determined by both the input image *x* and time interval *N*.

#### Loss network

Our study utilizes a loss network based on the popular VGG19 model [39], which was pretrained on over a million images from the ImageNet database [40]. This model has been shown to effectively capture both low-level and high-level features of images [26]. The features extracted from the loss network are then fed into various loss functions to calculate the differences between the content and style representations, ultimately guiding the optimization process during the training of the generative network.

As demonstrated in a series of works [28,29], the content similarity between 2 images can be obtained by calculating the Euclidean distance between their high-level features. In contrast, the style similarity needs to be measured by computing the similarity of lower-level features [26]. Because the low-level features contain too much spatial information, the Euclidean distance, which can be easily affected by the fruit size, location, and background in the images, is therefore not an ideal candidate for the style similarity measurement. Here, the Gram matrix of the feature maps is adopted to calculate the style correlation between different layers. This removes the influence of image content while retaining the information of low-level features. The network contains 5 convolution blocks each followed by a max pooling layer. Denoting the *j*-th convolution layer in the *i*-th convolution block as Conv*i*_*j*, Conv4_2 and Conv5_2 are used to calculate the content loss here, while Conv1_1, Conv2_1, Conv3_1, Conv4_1, and Conv5_1 are adopted to compute the style loss.

The height, width, and number of channels of the feature map after the *l*-th layer are denoted by *H_l_*, *W_l_*, and *C_l_*, respectively. Moreover, $x_{\mathrm{ijk}}^{l}$ represents the value at (*i*, *j*, *k*), which is correspondingly the 3-dimensional coordinates. The content loss is then formulated as:

$$L_{\text{content}}\left(x,\hat{y}\right)=\sum_{l}‍\frac{1}{H_{l}W_{l}C_{l}}\sum_{i,j,k}‍{\left(x_{\mathrm{ijk}}^{l}-{\hat{y}}_{\mathrm{ijk}}^{l}\right)}^{2}$$ (2)

After the *l*-th layer of convolution, the height–width matrix on the *k*-th channel of feature maps *x^l^* is flattened into a vector *F*^*l*,*k*^ with the length of *H_l_W_l_*. $F_{t}^{l,k}$ denotes the *t*-th element in *F*^*l*,*k*^, and the elements of the *m*-th row and *n*-th column of the Gram matrix can be calculated as:

$$G^{l}{\left(x\right)}_{\mathrm{mn}}=\sum_{t}‍F_{t}^{l,m}F_{t}^{l,n}$$ (3)

To compute the style difference only in the fruit region, the mask obtained from the semantic segmentation is used in the perceptual loss. For different layers, the mask is resized to the same size as the output feature map. Here, the ground truth is denoted as *y*, and *M^l^*(*y*) represents the mask of the *l*-th layer of *y*. Meanwhile, to reduce the influence of the fruit’s size change between the images, the Gram matrices are divided by $\sqrt{\sum ‍M^{l}\left(y\right)}$. The local style loss $L_{\text{style}}^{*}$ between 2 images is therefore defined as:

$$L_{\text{style}}^{*}\left(y,\hat{y}\right)=\sum_{l}‍\frac{W_{\text{style},l}}{{\left(H_{l}W_{l}C_{l}\right)}^{2}}\sum_{m,n}‍{\left(\frac{G^{l}{\left({\hat{y}}^{l}M^{l}\left(\hat{y}\right)\right)}_{\mathrm{mn}}}{\sqrt{\sum ‍M^{l}\left(\hat{y}\right)}}-\frac{G^{l}{\left(y^{l}M^{l}\left(y\right)\right)}_{\mathrm{mn}}}{\sqrt{\sum ‍M^{l}\left(y\right)}}\right)}^{2}$$ (4)

where *W*_style,*l*_ are the layer weight parameters. To ensure the fidelity of the generated image $\hat{y}$, it is expected to have a low local style loss with the ground truth *y*, as well as a low content loss with the input image *x*. Therefore, the local perceptual loss is defined as:

$$L_{\text{perceptual}}\left(x,y,\hat{y}\right)=L_{\text{content}}\left(x,\hat{y}\right)+L_{\text{style}}^{*}\left(y,\hat{y}\right)$$ (5)

## Results

In this section, the evaluation metrics are first introduced, and the experimental results of the semantic segmentation network, generative network, and sensory evaluation are then presented.

### Evaluation metrics

A commonly used metric, the Mean Intersection over Union (MIoU) [41], is adopted for segmentation evaluation. For the color prediction and visualization task, peak signal-to-noise ratio (PSNR) [42] is used to evaluate the quality of the generated image. A higher PSNR between the generated image and the original one indicates less distortion and higher image quality. The mean local style loss (MLSL) of the predicted result is computed as a metric of accuracy, and a low MLSL indicates a high similarity between the predicted citrus color and ground truth. In addition, the CCI of the predicted result and the ground truth are calculated, and CCI error is defined as the L1 distance between them. After converting the average RGB value from all the pixels of citrus semantic into HunterLab, the CCI is formulated as:

$$\mathrm{CCI}=\frac{1000\times a}{L\times b}$$ (6)

where *L*, *a*, and *b* are the coordinates of the HunterLab color space. Low CCI error indicates the high average color similarity between the predicted result and the ground truth.

### Semantic segmentation

**Fig. 3.**
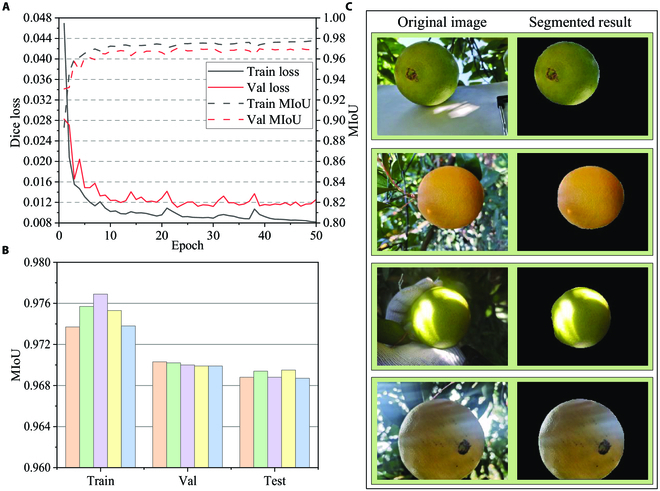
The training process and results of the semantic segmentation task. (A) The trend of Dice loss on the train set and validation set during training. (B) The performance of MIoU on different sets. (C) The input images (left) and their segmentation results (right).

The algorithm was implemented on a desktop with Intel(R) Xeon(R) Gold 6330 CPU @ 2.00GHz, NVIDIA GeForce RTX 3090, and 24 GB of video memory. We used Adam as the optimizer, along with the initial learning rate of 0.001, the first-order moment estimation of 0.9, the second-order moment estimation of 0.99, and the batch size of 56. Although the epoch was set to 50, the optimal MIoU of the model was obtained at the 23rd epoch on the validation set. The model training took about 40 min. The loss and MIoU on the training set and validation set during the training process are shown in Fig. 3A and B, respectively.

We selected 5 epochs with the highest MIoU values on the training set for testing. The MIoU on the test set achieved 0.969, demonstrating the capacity of the network to segment citrus images with different colors, backgrounds, and light intensities, as shown in Fig. 3C. As the performance is still satisfactory even when encountering strong or irregular light, good robustness can be ensured by the network model.

### Local style loss function

As a proper style loss function is vital for the training of generative networks, a comparative study was first conducted to obtain the optimal one. The style loss proposed by Gatys et al. [26] is a common choice, which is formulated as:

$$L_{\text{style}}\left(y,\hat{y}\right)=\sum_{l}‍\frac{1}{{\left(H_{l}W_{l}C_{l}\right)}^{2}}\sum_{m,n}‍{\left(G^{l}{\left({\hat{y}}^{l}\right)}_{\mathrm{mn}}-G^{l}{\left(y^{l}\right)}_{\mathrm{mn}}\right)}^{2}$$ (7)

**Fig. 4.**
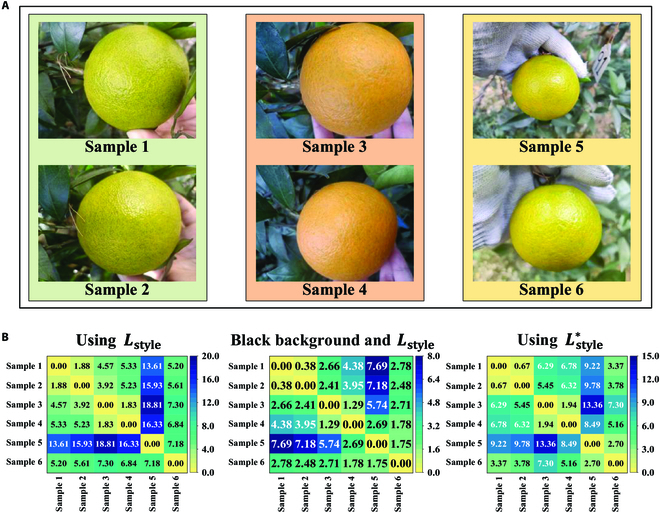
Selection of the style loss function. (A) The selected 3 sets of real images for the experiments, and each set consisted of 2 images with high similarity. (B) The style differences calculated in 3 different ways. The score in the *i*-th row and the *j*-th column represents the style difference between the *i*-th sample and the *j*-th sample. Each matrix is divided into 5 levels based on its highest and lowest values.

However, the performance of networks trained with it can be easily influenced by the target size and environmental aspects. In more recent work, the target was segmented and then placed in a black background, which helps reduce the influence of the original background [43]. To compare the effectiveness of the aforementioned style loss functions and our proposed one on challenging image sets, 3 sets of real images were first manually selected. Each set consisted of 2 images acquired with different orange sizes and backgrounds, but the oranges shared high similarity in color, light conditions, and texture characteristics based on human perception, as shown in Fig. 4A. The style differences of each 2 among these 6 images were then computed in 3 different ways, including:

1. *L*_style_: using *L*_style_ as the metric.
2. Black background and *L*_style_: multiplying the citrus image matrices with the masks to get the fruit with black background (pixel value is 0) and then using *L*_style_ as the metric.
3. $L_{\text{style}}^{*}$: using $L_{\text{style}}^{*}$ proposed in this work as the metric.

Figure 4B summarizes the obtained results, which are divided into 5 levels according to the maximum and minimum values of each matrix. For the first evaluation metric using *L*_style_, the results are generally consistent with human perception except for the similarity evaluation between samples 5 and 6. Because the oranges in the images closely resemble each other, the high *L*_style_ could be induced by the background information, as well as the size of the fruit. For the second evaluation metric, the same score was obtained when calculating the color difference between samples 4 and 5 and between samples 5 and 6, which also goes against human perception. This indicates that, despite its capacity to decrease the influence of the background, the effectiveness of the second metric is still affected by the size of the fruit. Our proposed metric outperforms those metrics in the extreme case, maintaining high consistency compared with human perception, and it was therefore adopted as the style loss function for the training of the generative network.

### Citrus color prediction and visualization

**Fig. 5.**
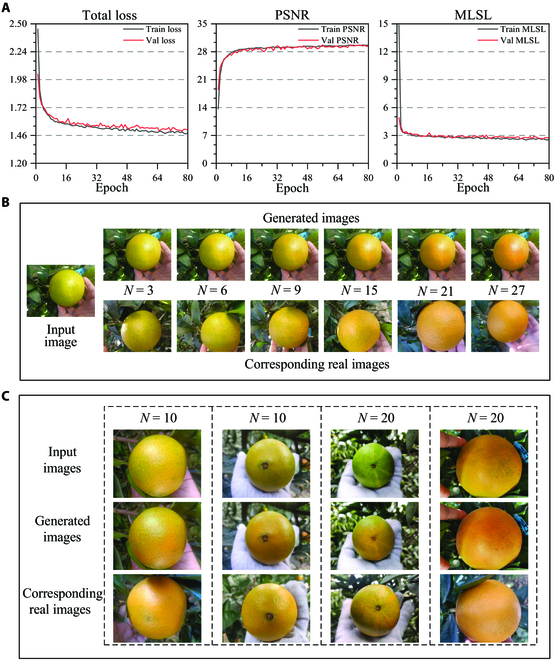
(A) The training process of the generative network. (B) The results generated from the same input image on the test set. *N* represents the input time interval. (C) The results generated from different input images on the test set.

For the task of citrus color prediction and visualization, the algorithm was implemented on an AMD EPYC 7543 32-Core Processor and NVIDIA A40, with 48 GB of video memory. The batch size was set to 32 and the epoch was set to 80. The model training took about 4 h. The total loss, PSNR, and MLSL on the training set and validation set during the training process are shown in Fig. 5A. To verify the robustness and generalization of the network, we first selected 10 models with the best MLSL performance on the validation set, among which 5 models with the best PSNR performance were then chosen for the experiments on the test set. As shown in Table 1, the highest PSNR obtained on the test set was 30.01, indicating good quality of the generated images, and the lowest MLSL achieved was 2.710, which demonstrates high similarity between the generated images and ground truth. In the 80th epoch, the CCI error dropped to 0.841, indicating that the average color of the predicted results was close to the ground truth.

**Table 1.** The results of generative network evaluation. The boldface numbers indicate the best performance achieved on the test set.

Epoch	PSNR			MLSL			CCI error		
	Train	Val	Test	Train	Val	Test	Train	Val	Test
69	29.614	29.060	29.858	2.583	2.744	2.814	0.859	1.043	1.057
73	29.577	29.486	29.323	2.569	2.848	2.735	0.845	1.055	0.994
78	29.661	29.617	29.751	2.550	2.774	2.728	0.826	0.987	0.955
79	29.611	29.363	**30.010**	2.507	2.757	**2.710**	0.788	1.004	0.933
80	29.683	29.349	29.837	2.592	2.717	2.719	0.814	0.959	**0.921**

Figure 5B shows the results when inputting a citrus image with different time intervals, and the color transformation process can be well predicted and visualized. As the time interval increases, the rind color of the fruit gradually deepens from yellow-green to orange, following the same trend as real images. Moreover, the brightness, texture characteristics, and color variations across different rind regions in the generated images also assemble the input image. Compared with one uniform color on the rind, such rich features indicate natural light conditions and are consistent with the background, which makes the generated images more realistic.

To further verify the robustness of the network model, experiments were conducted when inputting images of oranges with different viewing angles and colors, and the generated images still well replicate the color transformation compared with the ground truth, as shown in Fig. 5C. Note that uneven coloration on the orange rind, such as yellow stripes and local green spots, exists on the input images, and these features are also maintained on the generated images.

The embedding layers fused the image features and temporal information for the subsequent generation. To compare the performance of the merged model with individual models, models without embedding layers were trained at the time interval of 5/10/15/20 d, respectively. Their performance on the test set is shown in Table 2. In general, the proposed method could achieve higher PSNR while the individual models achieved the slightly lower MLSL and CCI error, and the differences are not significant. However, for the models without embedding layers, a set of model weights is needed to be stored for every time interval. The main advantage of our proposed method is that only one set of model weights needs to be trained, saved, and used for all kinds of time intervals.

**Table 2.** The comparison of the merged model and individual models.

Interval (d)	Model	PSNR	MLSL	CCI error
*N* = 5	With embedding layers	30.131	2.570	0.794
	Without embedding layers	29.638	2.443	0.739
*N* = 10	With embedding layers	29.892	2.767	1.003
	Without embedding layers	29.163	2.542	0.778
*N* = 15	With embedding layers	29.905	2.790	1.107
	Without embedding layers	29.735	2.414	0.717
*N* = 20	With embedding layers	29.527	2.612	0.933
	Without embedding layers	29.694	2.593	0.744

### K-fold cross-validation

To test the stability and effectiveness of our proposed approach, the K-fold cross-validation method was used for repetitions of the experimental evaluation. In this work, *k* was taken as 5. The dataset was evenly and randomly divided into 5 subsets based on different samples. Four subsets were used for training and one for testing. The experiment was repeated 5 times, with each subset tested once. The results is shown in Table 3. Each test set achieved IoU of over 0.969 and PSNR of over 28.837. The average values of MLSL and CCI error on the test set were 2.685 and 0.923, respectively.

**Table 3.** The result of K-fold cross-validation. The boldface numbers indicate the best performance achieved on the test set.

Fivefold	Set	IoU	PSNR	MLSL	CCI error
5-1	Train	0.976	28.989	2.597	0.753
	Test	0.969	29.573	**2.612**	**0.861**
5-2	Train	0.974	29.569	2.631	0.872
	Test	0.971	28.837	2.721	0.979
5-3	Train	0.975	29.970	2.674	0.907
	Test	0.970	**29.869**	2.703	0.926
5-4	Train	0.977	30.004	2.689	0.899
	Test	0.969	29.841	2.751	0.977
5-5	Train	0.976	29.527	2.611	0.852
	Test	**0.972**	29.641	2.639	0.871

### Sensory evaluation

**Fig. 6.**
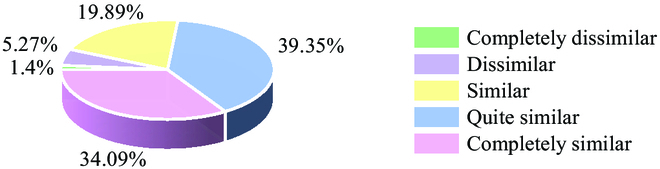
The result of sensory evolution regarding the similarity between the synthesized image and the real one.

Specialist sensory panels also assessed the perceptual realism and accuracy of the generated pictures. The sensory panels averaged 31 participants sampled from the scientists at National R&D Center for Citrus Preservation, Wuhan, China, and informed consent was provided by all participants. A total of 30 pairs of images were randomly adopted for the sensory evaluation. Each pair consisted of a synthesized image generated by our proposed method and the corresponding real image. The panels then assessed the similarity between these images in each pair using a 5-point scale, in which 5 denoted “Completely Similar” and 1 corresponded to “Completely Dissimilar.” The results were statistically analyzed as shown in Figure 6. Only 6.67% of the image pairs were rated as “Dissimilar” and below, indicating good perceptual realism and accuracy between the synthesized image and the real one. Note that our method does not require strict structure similarity when acquiring images, which can also cause perceptual differences.

## Discussion

Overall, our model achieves a good semantic segmentation performance of the citrus images, along with the accurate prediction of citrus color transformation at different time intervals. To the authors’ knowledge, we present the first framework to realistically visualize the prediction of citrus color transformation in the orchard. Compared with presenting only a color parameter such as CCI, generated images provide rich prototyping features such as shape, color variations, and texture properties, which attain great importance for the monitoring of fruit development and are therefore very useful for horticulture research. Moreover, because of the high correlation between the citrus rind color and maturity stage, the predicted results also assist in the determination of the optimal harvest time, and visualizing helps to clearly deliver the information to those even with no prior knowledge. While Navel oranges were taken as the sample fruit in this study, the workflow presented can be readily expanded to other citrus species if associated with a color transformation period. Note that color transformation can also be influenced by environmental conditions such as soil composition, temperature, and humidity, and these aspects can be addressed by using the embedding layers in our model. Although there still lacks systematic research on the influence of environmental effects on citrus fruit development, the duration of the color transformation period is relatively constant each year based on experience. However, a sudden decrease in temperature could significantly accelerate this process. The method’s applicability to other varieties as well as in even earlier and later maturity stages is also worth further exploring, and model performance could be potentially improved if more information is introduced via embedding layers.

**Fig. 7.**
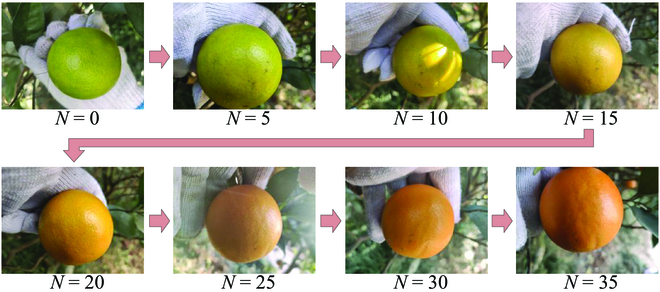
The input image and ground truth.

Edge-based application of GAN in the orchard is challenging because of 2 major reasons: (a) high training costs and a large number of model parameters, requiring a lot of computation and storage resources, and (b) strict similarity in the structure of the acquired images, which can be hardly achieved in the natural environment. For the former issue, we manually designed a series of loss functions instead of training a discriminator, thus significantly lowering the computational complexity. Moreover, the introduction of embedding layers in the generative network fused the information of the input image and time interval *N*. As a result, instead of training one model for each time interval, only one model is needed here to predict color transformation at different time intervals, and model parameters and required storage resources can therefore be greatly reduced. For the latter issue, because of the proposed style loss function $L_{\text{style}}^{*}$, one-to-one correspondence at the pixel level between the input image and ground truth is no longer needed as shown in Fig. 7, thus easing the process of data acquisition, and better image quality and color prediction accuracy can also be obtained according to the experiment results.

**Fig. 8.**
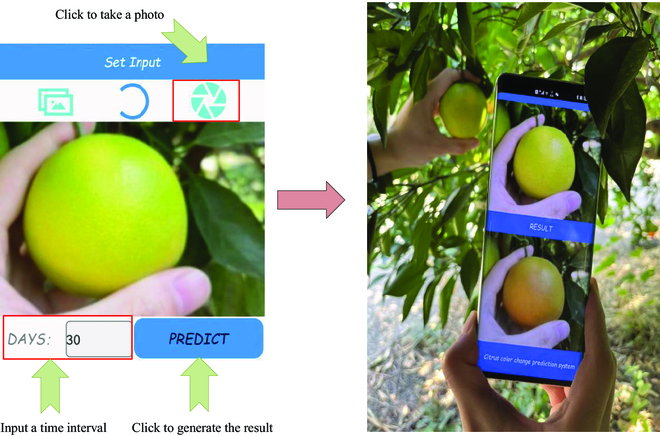
The testing of the Android-based application.

Owing to aforementioned advantages, the network is suitable for edge computing, and we therefore ported the trained model to an Android smartphone with a built-in camera, as shown in Fig. 8. The citrus image can be then acquired using the device, and the the future image of the fruit can be generated if an interested time interval *N* is provided. The predicted color information is presented in a visualized manner, with which the maturity stage can be more easily approximated. Meanwhile, better arrangement can also be attained for the crop management practices and harvest schedule.

The complete pipeline demonstrates the feasibility of using a generative model to predict and visualize the color transformation of plant parts. While our work was conducted in the field, the proposed model has proven to perform robustly in complicated environments. Therefore, applications can also be found in the warehouse to better monitor the ripeness of certain types of fruits, such as bananas and mangoes, which is vital for their post-harvest management. Moreover, the model might serve as a tool to analyze the influence of environmental aspects on fruit development when taking them into account. Furthermore, methods to predict and visualize the transformation of other phenotyping characteristics can be potentially developed following the pipeline, thus assisting and accelerating germplasm research. These issues will be addressed in future work.

### Conclusion

This study presents the complete workflow to predict and visualize citrus color transformation in the orchard. A dataset was built containing citrus images through the color transformation period, which was used for network training and validation. A framework based on a deep mask-guided generative network was developed to first segment the fruit in the image, and the future rind color was then predicted in a visualized manner. Moreover, a series of loss functions was designed to replace the discriminator, which significantly decreased the computation costs of network training. The fusion of image features and temporal information also enabled one single model to predict the rind color at different time intervals, thus effectively shrinking the number of model parameters. Experimental results demonstrated the high accuracy and robustness of the network, and the generated images were consistent with human perception featuring good fidelity. The merged design of the network made facilitated easy implementation on mobile devices, and an Android-based application was developed for in-field applications. The presented methods can be readily expanded to different citrus species and other fruit crops if associated with a color transformation period.

## Data Availability

The data used in this paper will be available upon request here: https://github.com/BaoZehan/DeepCCP.
